# Supplementary material for: GPX2 Gene Affects Feed Efficiency of Pigs by Inhibiting Fat Deposition and Promoting Muscle Development
Source: Animals (Basel). 2022 Dec 14;12(24):3528. doi: 10.3390/ani12243528 (PMC9774625; doi:10.3390/ani12243528)
Supplement: Supplementary file 1 [file animals-12-03528-s001.zip › Supplementary figure.pdf]

A

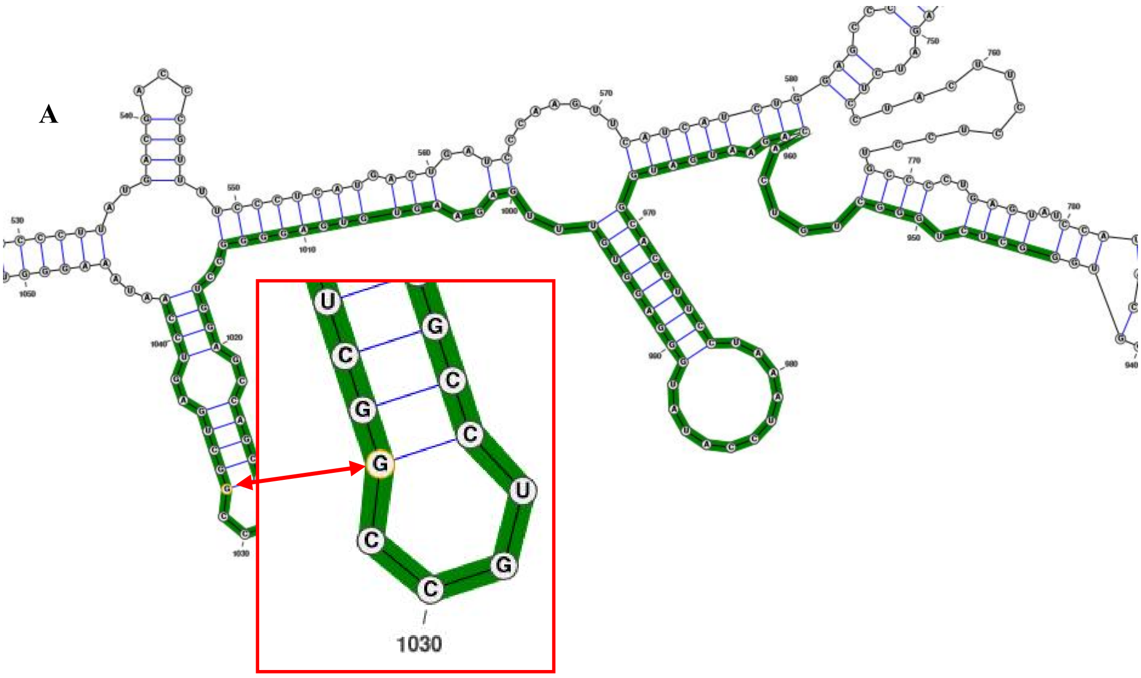

B

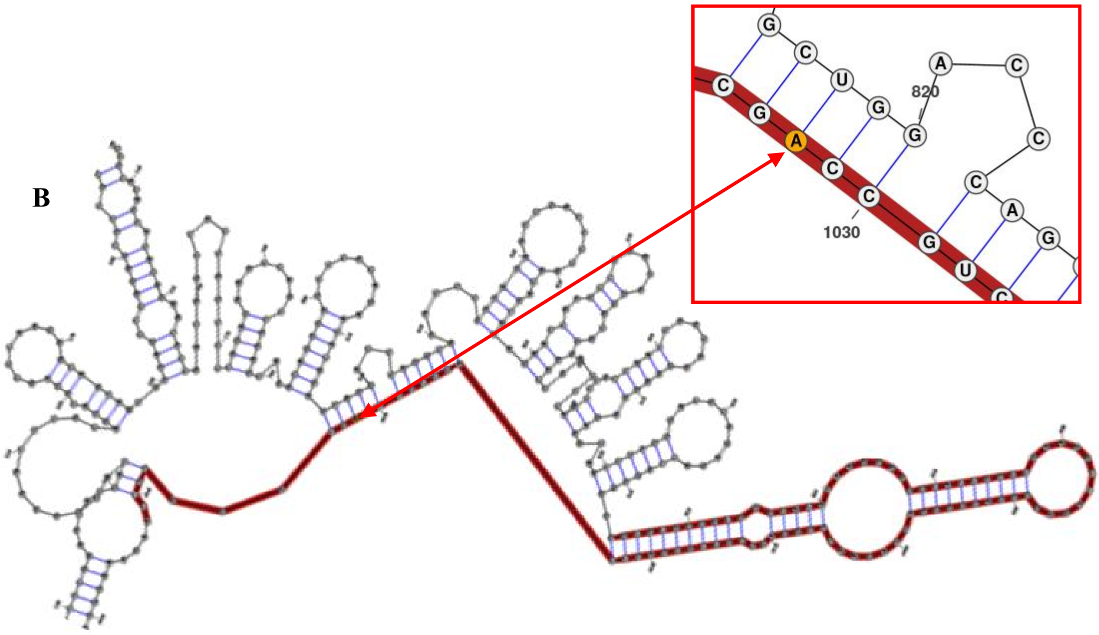

Supplementary Figure S1 Planar polygon diagram of c.1032 G > A of *GPX2* effect on local RNA secondary structure. **(A)** The optimal secondary structure of the wild-type sequence, minimum free energy = -346.60 kcal/mol, the green portion was the local RNA secondary structure on 944-1042 bp. **(B)** The optimal secondary structure of the mutant sequence, minimum free energy = -338.60 kcal/mol, the red portion was the local RNA secondary structure on 944-1042 bp. The larger version of 1032 was in the red box.

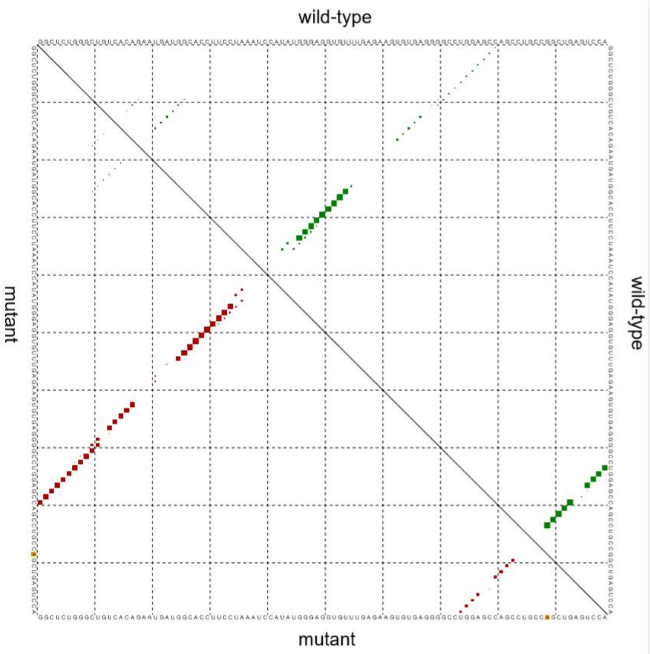

Supplementary Figure S2 Screw the lattice diagram of c.1032G>A of *GPX2* effect on local RNA secondary structure.

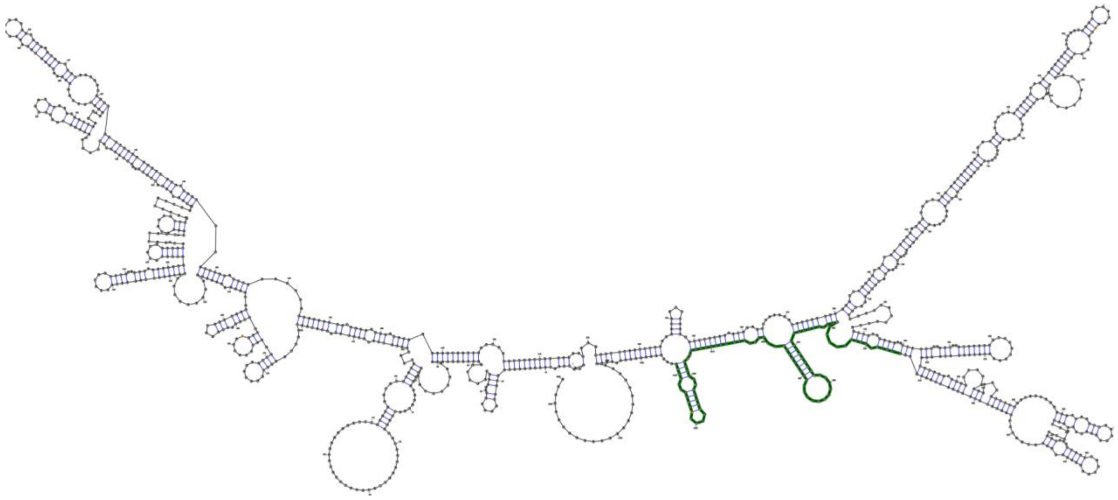

Supplementary Figure S3 Planar polygon diagram of c.1032G>A of *GPX2* effect on local RNA secondary structure of global wild-type sequence.

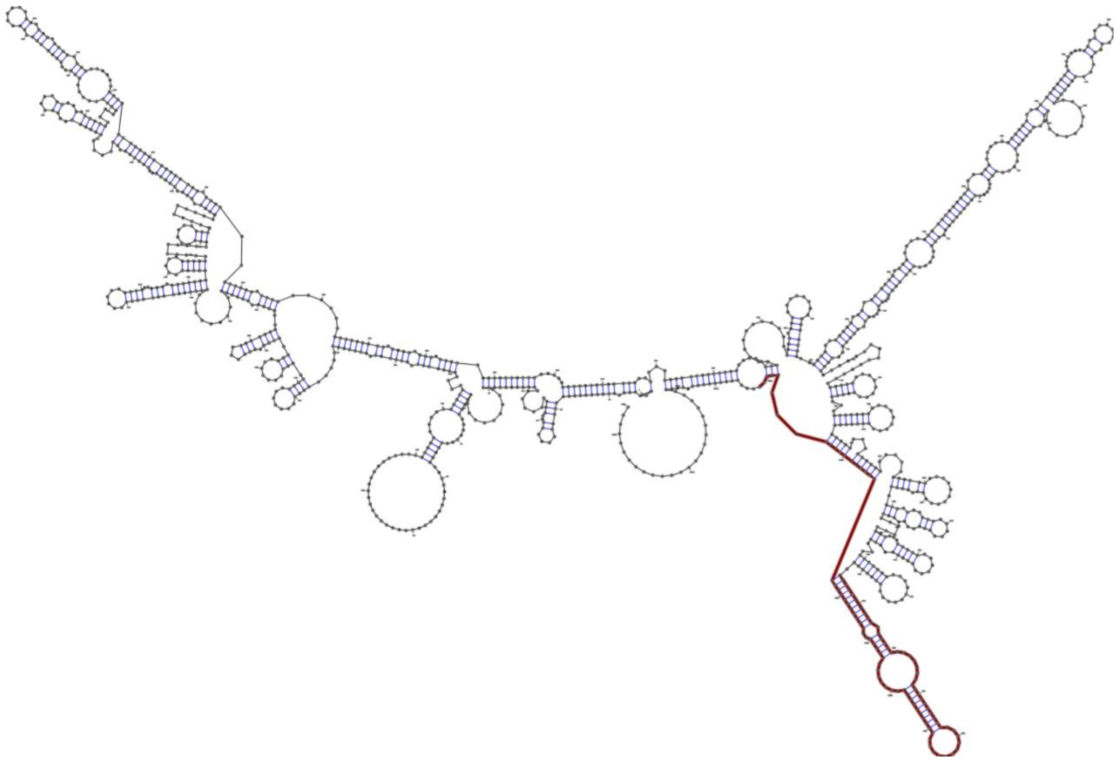

Supplementary Figure S4 Planar polygon diagram of c.1032G>A of *GPX2* effect on local RNA secondary structure of mutant-type sequence.
