# Supplementary material for: GPX2 Gene Affects Feed Efficiency of Pigs by Inhibiting Fat Deposition and Promoting Muscle Development
Source: Animals (Basel). 2022 Dec 14;12(24):3528. doi: 10.3390/ani12243528 (PMC9774625; doi:10.3390/ani12243528)
Supplement: Supplementary file 1 [file animals-12-03528-s001.zip › Supplementary table.pdf]

Supplementary Table S1 Primers used for amplification of *the* 5′ flanking region and exons for *GPX2* gene

**Primers**	**Primer sequences(5′~3′)**	**Product size (bp)**	**Annealing T(℃)**	**Amplification target**
EF1	GGGGCAAGTAAGAGTCA	645	53.5	Exon1
ER1	CCCTTAGCCCACTAGAAC			
EF2	CCCTTGTCCAAACTCTGT	428	53.5	Exon2-1
ER2	GGCAGGAGGAAGTAGGA			
EF2	CCCCACCATCAACATCGA	586	53.5	Exon2-2 and 3'UTR
ER2	CCTATGACCAATCGCAGA			
5′FF1	CCCAGAAGGGCAGCCAGT	701	53.5	5′ flanking region 1
5′F R1	CCTGCTAAGGGCTATTGT			
5′F F2	CCTGTGAAAGCACCATCT	532	53.5	5′ flanking region 2
5′F R2	ACTGTACCGTCTCCTCTAA			
5′F F3	GGGGCATTAGAGGAGACG	742	53.5	5′ flanking region 3
5′F R3	GGAGGGATGCAGGATTGA			
GPX2F	CCCCACCATCAACATCGA	96	60.0	qRT-PCR
GPX2R	GGCAGGAGGAAGTAGGA			
GAPDH	AGGGCATCCTGGGCTACA	166	60.0	qRT-PCR
GAPDH	TCCACCACCCTGTTGCTGT			

Supplementary Table S2 Primers and probes for genotyping assay for seven SNPs of *GPX2* gene

**Primers**	**Primer sequences (5′~3′)**	
c.1032G>A	Forward	ACGTTGGATGTCCACACCCACCCTTTATTG
	Reverse	ACGTTGGATGCCATATGGGAGGTGTTTGAG
	Probe	CCACCCTTTATTGGACTCAGC
c.665T>C	Forward	ACGTTGGATGTTGAGAAGTTCCTCATCGGG
	Reverse	ACGTTGGATGTGTCAGGCTCGATGTTGATG
	Probe	CTTCCGACGCTACAG
c.182C>T	Forward	ACGTTGGATGGCACTTCCACCATGGCTTAC
	Reverse	ACGTTGGATGAAATCTACCTTCTCCCCGTC
	Probe	ATTGCCAAGTCCTTCTA
g.-774T>G	Forward	ACGTTGGATGCTTTAGGAGGATGGTGTGTG
	Reverse	ACGTTGGATGTTTACCTCTGTAGCAGGATG
	Probe	GAGTATGGTGTGTGATTTTTCTAA
g.-1043C>T	Forward	ACGTTGGATGACAAGCCCCTCTAGAAAGTC
	Reverse	ACGTTGGATGGTACAAGTGGTAAACTCGGC
	Probe	CCTCTAGAAAGTCTTTAGGA
g.-1333G>A	Forward	ACGTTGGATGAAGGTTTCTGTCTGTGTGTG
	Reverse	ACGTTGGATGAGCCAGGTAAAGCCTAATCG
	Probe	CCACCTGCCTGGATTTCTCCATC
g.-1531G>C	Forward	ACGTTGGATGACTTAGGTAAGGGAGGCTTG
	Reverse	ACGTTGGATGGTGGCTGTTTATAGCAACGC
	Probe	GTAGCCAGTTGAGACC

Supplementary Table S3 primer sequences of qRT-PCR SNP

primer name	primer sequence(5'-3')	product size/bp
GPX2 F	CCTGGATGGGGAGAAGA	158
GPX2 R	AAGGGAAGCCGAGAACTA	
mGPX2 F	CCCCACCATCAACATCGA	251
mGPX2 R	GGCAGGAGGAAGTAGGA	
Cyclin B F	TCTTGCTTGGCTTCATTCATAG	124
Cyclin B R	TTCTTTTCCAGGTGGCATTAC	
CDK2 F	ACTCTTGGTCTGTTCATCGTGGT	109
CDK2 R	GCAAAGTCCTGGGTGTAGCA	
P21 F	GGTGGTGGAGACCTGATGAT	108
P21 R	ATTCTGCTGGCAAAGTGGGA	
PPARγ F	GACGCGGAAGAAGAGACCTG	84
PPARγ R	TCACCTTGTCGTCACACTCG	
AP2 F	CCTTTGTGGGAACCTGGAA	225
AP2 R	TGTCGTCTGCGGTGATTT	
FAS F	GGGTCTATGCCACGATTC	272
FAS R	GTGTCCCATGTTGGATTTG	
CEBPα F	GCAAAGCCAAGAAGTCGGTG	145
CEBPα R	TCACTGGTCAACTCCAGCAC	
HSL-F	AAGACCACATCGCCCACAGC	159
HSL-R	GCTGTCTGAAGGCTCTGAGTTGC	
ATGL-F	TGCTGGAGGCCTGTGTGGAA	160
ATGL-R	TCAGGGACATCAGGCAGCCACT	
LPL-F	TGGCGTAGCAGGAAGTCTGA	265
LPL-R	TGCAATCACACGGATGGC	
MyoG F	GACCCTACAGACGCCCACAA	60
MyoG R	CCGTGATGCTGTCCACGAT	
MyoD F	CGGCTCTCTCTGCTCCTTTG	60
MyoD R	GTCGAAACACGGGTCATCA	
MYST F	TGTAACCTTCCCAGAACCAG	269
MYST R	GCAATAATCCAATCCCATCC	
MYH3 F	CCACCTGAACGAGCCC	207
MYH3 R	TGGTAGGCGTTGTCGG	
GAPDH F	AGGAGAGTGTTTCCTCGTCC	187
GAPDH R	TGCCGTGAGTGGAGTCATAC	

Supplementary Table S4 Statistical description of data set for feed efficiency related traits in 383 Durocs

**Trait**	**Mean**	**SD[1]**	**Max[2]**	**Min[3]**	**CV (*100%)[4]**
Birth weight（kg）	1.79	0.30	2.74	0.84	0.17
Weaning weight（kg）	7.76	1.96	13.40	3.92	0.25
90d BW（kg）	29.51	5.55	45.80	16.20	0.19
ADFI（kg）	1.70	0.27	2.44	1.05	0.16
ADG（kg）	0.65	0.12	0.97	0.27	0.19
30kg age（d）	90.77	8.63	116.80	65.30	0.10
100kg age（d）	196.45	15.17	241.56	164.58	0.08
100kg BF（mm）	7.42	1.34	12.44	4.06	0.18
FCR	2.66	0.35	3.87	2.09	0.13
RFI（g）	3.27	135.40	486.72	-583.82	41.41

[1]SD: standard deviation; [2]Max: maximum value; [3]Min: minimum; [4]CV: coefficient of variation; 90 d BW: body weight at 90 d of age; ADFI: average daily feed intake; ADG: average daily gain; 100kgBF: back fat at 100 kg; FCR: feed conversion ratio; RFI: residual feed intake.

Supplementary Table S5 Statistical description of data set for feed efficiency related traits in 600 Durocs

Trait	Mean	SD[1]	Max[2]	Min[3]	CV (*100%)[4]
Birth weight（kg）	1.79	0.30	2.74	0.84	0.17
Weaning weight（kg）	7.98	1.67	14.34	3.92	0.21
90d BW（kg）	29.48	5.10	45.80	16.20	0.17
ADFI（kg）	1.69	0.26	2.44	1.00	0.15
ADG（kg）	0.66	0.12	1.04	0.27	0.18
30kg age（d）	90.80	7.84	116.80	65.30	0.09
100kg age（d）	195.06	14.66	241.56	157.84	0.08
100kg BF（mm）	7.74	1.43	13.10	4.05	0.18
FCR	2.59	0.36	3.90	1.74	0.14
RFI（g）	9.42	158.37	494.30	-583.82	16.81

[1]SD: standard deviation; [2]Max: maximum value; [3]Min: minimum; [4]CV: coefficient of variation; 90 d BW: body weight at 90 d of age; ADFI: average daily feed intake; ADG: average daily gain; 100 kg BF: back fat at 100 kg; FCR: feed conversion ratio; RFI: residual feed intake.
